# Altered mental status in cancer-related emergency hospitalizations: prevalence and recorded reasons for admission

**DOI:** 10.1186/s12873-026-01667-9

**Published:** 2026-07-06

**Authors:** Lindun Ge, Yasufumi Gon, Yasuyoshi Kimura, Toshitaka Morishima, Tatsusada Okuno, Hideki Mochizuki, Isao Miyashiro

**Affiliations:** 1 Department of Neurology, Graduate School of Medicine, The University of Osaka, Osaka, Japan; 2 Cancer Control Center, Osaka International Cancer Institute, Osaka, Japan; 3 Department of Neurology, National Hospital Organization Osaka Toneyama Medical Center, Osaka, Japan

**Keywords:** Altered mental status, Cancer-related emergencies, Emergency hospitalization, Japan coma scale, Cancer registry

## Abstract

**Background:**

Altered mental status is a critical clinical presentation in emergency departments, yet its frequency in cancer-related emergency hospitalizations and the clinical conditions recorded at presentation have not been well characterized.

**Methods:**

We conducted a retrospective multicenter cohort study using data from the Cancer Registry-based Study on Cancer Care in Osaka (CanReCO), which links hospital-based cancer registry data with Diagnosis Procedure Combination (DPC) administrative data. The cohort included patients diagnosed between January 2019 and December 2022, and linked DPC hospitalization records available through June 2023 were used to identify first emergency hospitalizations after cancer diagnosis. We extracted cases presenting with altered mental status, defined as a Japan Coma Scale score ≥ 1. Recorded reasons for admission were described overall and according to cancer stage and cancer type.

**Results:**

Of 55,949 cancer-related emergency hospitalizations across 68 participating hospitals, 5,881 (10.5%) involved altered mental status. The most frequent reasons for admission among patients with altered mental status were index cancer (32.3%), stroke (8.6%), and pneumonia (6.9%). When analyzed by cancer stage at diagnosis, index cancer, CNS metastasis, and sepsis were more frequently recorded in later stages. When analyzed by cancer type, certain reasons were more frequent in specific malignancies, including CNS metastasis in lung and breast cancers; pneumonia in lung cancer; sepsis in hematologic, pancreatic, and biliary cancers; and hyperglycemia or diabetes mellitus in pancreatic cancer, among others.

**Conclusions:**

Altered mental status was commonly recorded among cancer patients requiring emergency hospitalization. The reasons for admission varied by cancer stage and cancer type, with neurological and infectious diagnoses frequently observed. These findings provide descriptive information that may help clinicians consider cancer-specific patterns when evaluating cancer patients presenting with altered mental status in emergency settings.

**Supplementary information:**

The online version contains supplementary material available at https://doi.org/10.1186/s12873-026-01667-9.

## Introduction

The number of cancer patients has been steadily increasing worldwide, leading to a corresponding rise in cancer-related emergency department (ED) visits [1]. In the United States, cancer-related ED visits accounted for approximately 4.2 million annual visits [2], while in South Korea, a study analyzing ED utilization by adult cancer patients found that nearly one in ten cancer patients required emergency care during their disease course, comprising 5.5% of total ED visits nationwide [3]. The reasons for ED visits among cancer patients are diverse, ranging from disease-related complications to treatment-related toxicities. A nationwide study in the U.S. reported that the most common causes of cancer-related ED visits included infections, pain crises, respiratory distress, and complications related to chemotherapy [2]. Given the increasing burden of cancer-related ED visits, understanding the characteristics and primary clinical conditions associated with these visits is crucial for improving emergency oncology care.

On the other hand, altered mental status is a significant clinical presentation in general ED visits, requiring urgent evaluation and intervention [4]. Studies suggest that altered mental status accounts for approximately 0.9% to 5% of all ED presentations, though the prevalence varies by region and study population [5–9]. The underlying clinical conditions associated with altered mental status are highly variable, with studies identifying different commonly reported diagnoses depending on demographic factors and healthcare settings. A study in China identified neurological disorders (35.0%) and toxicological causes (23.0%) as the most common diagnostic categories among ED patients with altered mental status [6], while a multicenter study in South Korea found that metabolic disorders (24.7%) and systemic infections (17.3%) were the leading factors [7]. In contrast, a German study examining admitted cases of altered mental status found that cerebrovascular diseases (24%), infections (14%), epileptic seizures (12%), and psychiatric diseases (8%) were the most frequent diagnoses [8]. The variability in reported diagnostic patterns underscores the importance of understanding local and population-specific trends in altered mental status. Given its broad differential diagnosis and potential severity, characterizing the clinical conditions associated with altered mental status in different patient populations is critical for guiding diagnostic evaluation and management in the ED [9].

Despite extensive research on both cancer-related emergencies and altered mental status in ED settings, the intersection of these two domains remains underexplored. A single-center study from Brazil reported that altered mental status was a notable presenting symptom among cancer patients admitted through the ED [10], highlighting the need for further investigation into this topic. However, there is limited population-based evidence describing how frequently altered mental status occurs among cancer-related emergency hospitalizations and what clinical conditions are recorded at the time of admission in these patients.

Therefore, this study aims to investigate the prevalence of altered mental status among cancer-related emergency hospitalizations using a multicenter cancer registry linked to administrative data. The primary outcomes are the proportion of emergency hospitalizations with altered mental status and the recorded reasons for admission among these patients. We further examine whether these patterns vary according to cancer stage and cancer type.

## Materials & methods

### Standard protocol approvals and patient consent

This study complied with the Declaration of Helsinki and was approved by the Institutional Review Board of University of Osaka Hospital (approval number: 22,485). Because of the retrospective nature of the study and the availability of an opt-out opportunity on the institutional website, the requirement to obtain written informed consent was waived.

### Study design

We conducted a retrospective multicenter cohort study to investigate the prevalence of altered mental status and the recorded reasons for admission among cancer patients requiring emergency hospitalization, using data from the Cancer Registry-based Study on Cancer Care in Osaka (CanReCO) project. The CanReCO database was developed in collaboration with the Council for Coordination of Designated Cancer Care Hospitals in Osaka and comprises hospital-based cancer registry data linked with administrative data produced under Japan’s Diagnosis Procedure Combination (DPC) system. The cancer registry cohort included patients diagnosed between January 2019 and December 2022, and linked DPC hospitalization records available from January 2019 to June 2023 were used to identify emergency hospitalizations occurring after cancer diagnosis. Data were obtained from 68 designated cancer care hospitals (DCCHs) and former DCCHs across Osaka Prefecture, Japan [11–13]. The DPC system is a standardized patient classification framework implemented in 2003 by the Japanese government to enhance medical care standardization, quality assessment, and resource allocation [14]. The linkage of CanReCO and DPC data allowed analysis of cancer characteristics and emergency hospitalizations across participating hospitals.

### Data selection

**Fig. 1:**
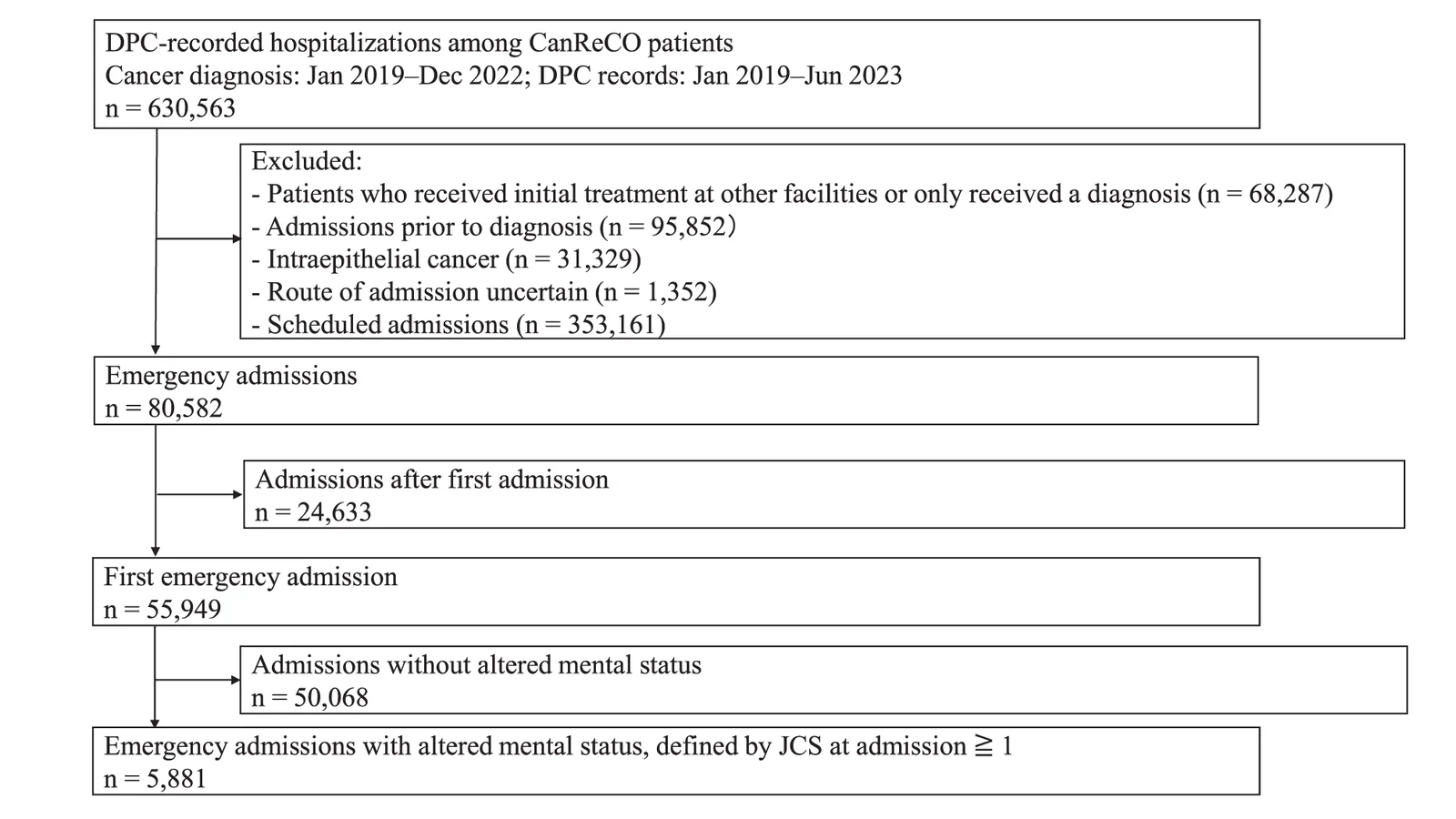
Flowchart of data selection. CanReCO, cancer registry-based study on cancer care in Osaka; DPC, diagnosis Procedure Combination; JCS, Japan Coma Scale

A total of 630,563 hospitalizations recorded in the linked DPC hospitalization data from January 2019 to June 2023 among patients in the CanReCO cohort were initially identified. From these, we excluded scheduled (non-emergency) cases, those with an uncertain admission route, and patients with intraepithelial (stage 0) cancer, as emergency admissions in patients with stage 0 cancer were considered unlikely to reflect cancer-related emergencies. We also excluded admissions that occurred before the patient’s cancer diagnosis and cases in which patients received their initial treatment at other facilities or were only registered as having a diagnosis without treatment information. After applying these criteria, 80,582 emergency admissions remained. To avoid duplication, only the first emergency hospitalization following cancer diagnosis for each patient was included, resulting in a final study population of 55,949 cases. Details are shown in Fig. 1.

### Clinical variables

The following variables were obtained from the CanReCO database: sex, age at cancer diagnosis, year of cancer diagnosis, stage at cancer diagnosis, cancer type, and duration from cancer diagnosis to emergency hospitalization. Age at cancer diagnosis was grouped into six categories: 0–19, 20–39, 40–59, 60–69, 70–79, and ≥80 years. The stage at cancer diagnosis was classified in accordance with the Union for International Cancer Control (UICC) TNM (“Tumor”, “Nodes”, “Metastases”) Classification of Malignant Tumors into five categories (stage I, II, III, IV, and unknown or not applicable) [15, 16]. Patients with stage 0 were excluded from the study population. The duration from cancer diagnosis to emergency admission was categorized into the following five groups: simultaneous, 1 day-1 month, 1 month-3 months, 3 months-1 year, and >1 year. Simultaneous indicates that cancer was diagnosed when the patient was hospitalized for another reason.

### Definition of altered mental status and recorded reasons for admission

Altered mental status was defined using the Japan Coma Scale (JCS), which is widely used in Japan for assessing consciousness and mental status in emergency settings [17]. The JCS categorizes consciousness impairment into four main levels: 0, alert consciousness; 1–3, awake without stimulation but not fully oriented; 10–30, arousable by verbal or pain stimuli; and 100–300, unresponsive to stimuli, with further subdivisions for granularity [17, 18]. Specifically, JCS 1 indicates that the patient is almost fully conscious but slightly confused, JCS 2 indicates disorientation to time, place, or person, and JCS 3 indicates inability to recall one’s name or date of birth. Previous studies have shown that JCS scores correlate with clinical outcomes, including in-hospital mortality and severity of neurological impairment [17–19]. Previous studies have discussed conversion between JCS and the Glasgow Coma Scale (GCS), but have noted that JCS and GCS should be assessed independently because their assessment components differ [19, 20]. Because the DPC system records JCS but not GCS, JCS was the only available measure of consciousness in this database. We defined altered mental status as JCS ≥ 1 to capture a broad spectrum of abnormal mental status recorded in the DPC system, ranging from mild confusion or disorientation to severe impairment of consciousness.

Recorded reasons for admission were extracted from the “reason for admission” field in the DPC system. Diagnoses in the DPC system are registered using International Classification of Diseases, 10th Revision codes, and their accuracy has been reported elsewhere [21]. The disease diagnosis codes used in this study are detailed in Supplemental Tables 1 and 2. In this study, “index cancer” was used when the recorded reason for admission corresponded to the patient’s registered primary cancer. This category may include hospitalizations related to symptoms, functional decline, or direct complications attributed to the cancer itself. However, because the DPC data do not provide detailed clinical adjudication, this category may also include cases in which a more specific immediate reason for admission was not determined in the ED and the known cancer diagnosis was recorded as the reason for admission.

### Statistical analysis

Baseline characteristics were summarized using counts with percentages for categorical variables.

We first calculated the proportion of first emergency hospitalizations after cancer diagnosis in which altered mental status was recorded at admission. We then described the distribution of recorded reasons for admission among all emergency hospitalizations and among those with altered mental status. Stratified descriptive analyses were performed according to cancer stage and cancer type to examine how the distribution of recorded reasons for admission varied across these categories. These analyses were descriptive and were not intended to assess causal relationships or effect modification. The frequencies of recorded reasons for admission were visualized using bar charts. All analyses were performed using the R software (https://cran.r-project.org/).

### Data availability

The data supporting this study’s findings can be obtained from the corresponding author upon reasonable request.

## Results

### Study population

The study included 55,949 patients who experienced their first emergency hospitalization following a cancer diagnosis (all emergency hospitalizations, AEH group). Among them, 5,881 patients (10.5%) presented with altered mental status at the time of admission (altered mental status, AMS group). Table 1 presents the baseline characteristics of patients in the AEH and AMS groups. The proportion of male patients was higher than that of female patients in both groups, with 60.7% in the AEH group and 59.2% in the AMS group. Regarding age at cancer diagnosis, patients aged 80 and above were more prevalent in the AMS group (46.6%) than in the AEH group (30.5%). The stage at cancer diagnosis differed slightly between the groups, but the frequency of Stage IV was almost the same. For time from diagnosis to emergency admission, the most common durations in both groups were 3 months to 1 year, with 35.4% in the AEH group and 35.3% in the AMS group.

**Table 1:** Baseline characteristics of the AEH and AMS groups

Baseline characteristics	AEH group (n,%)	AMS group (n,%)
Patients	55,949	5,881
Sex		
Male	33,969 (60.7)	3,483 (59.2)
Female	21,980 (39.3)	2,398 (40.8)
Age at diagnosis		
0–19	298 (0.5)	19 (0.3)
20–39	844 (1.5)	43 (0.7)
40–59	6,531 (11.7)	360 (6.1)
60–69	9,698 (17.3)	703 (12.0)
70–79	21,509 (38.5)	2,018 (34.3)
80-	17,069 (30.5)	2,738 (46.6)
Year of diagnosis		
2019	17,288 (30.9)	1,685 (28.7)
2020	12,685 (22.7)	1,263 (21.5)
2021	12,807 (22.9)	1,373 (23.3)
2022	13,169 (23.5)	1,560 (26.5)
Clinical stage at diagnosis		
Stage I	11,334 (20.3)	1,053 (17.9)
Stage II	7,133 (12.7)	673 (11.4)
Stage III	9,892 (17.7)	860 (14.6)
Stage IV	18,915 (33.8)	1,982 (33.7)
Unknown or not applicable*	8,675 (15.5)	1,313 (22.3)
Duration (diagnosis to admission)		
Simultaneous	12,218 (21.8)	1,573 (26.7)
1 day − 1 month	8,358 (14.9)	733 (12.5)
1 month − 3 months	11,211 (20.0)	1,017 (17.3)
3 months − 1 year	19,823 (35.4)	2,075 (35.3)
>1 year	4,339 (7.6)	483 (8.2)

*Hematologic malignancies cannot be categorized into UICC stages and therefore are labeled ‘not applicable’

AEH, all emergency hospitalization; AMS, altered mental status

### Recorded reasons for admission

**Fig. 2:**
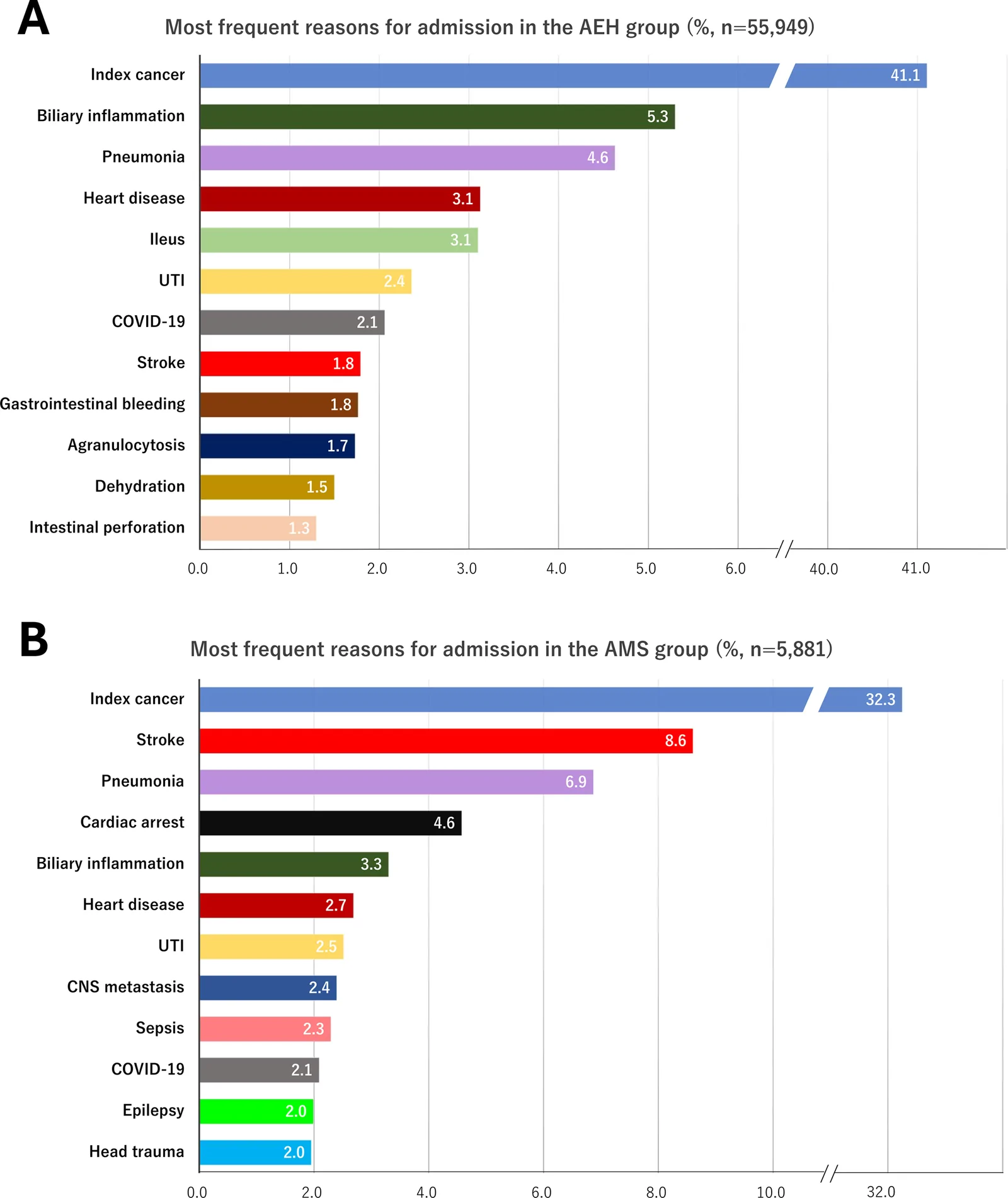
Most frequent reasons for admission in **A**) the AEH group and **B**) the AMS group. CNS, central nervous system; COVID-19, coronavirus disease 2019; UTI, urinary tract infection

Figure 2A illustrates the recorded reasons for emergency hospitalization in the AEH group. Index cancer was the most common reason for hospitalization (41.1%), followed by biliary inflammation (5.3%), pneumonia (4.6%), heart disease (3.1%), ileus (3.1%), urinary tract infection (2.4%), COVID-19 (2.1%), stroke (1.8%), and gastrointestinal bleeding (1.8%). Figure 2B displays the reasons for emergency hospitalization in the AMS group. Index cancer was again the most frequent recorded reason for admission (32.3%). Diagnoses involving the nervous system were more prevalent in the AMS group, including stroke (8.6%), CNS metastasis (2.4%), epilepsy (2.0%), and head trauma (2.0%). Infections such as pneumonia (6.9%), biliary inflammation (3.3%), urinary tract infection (UTI) (2.5%), and COVID-19 (2.1%) were also common. Cardiac arrest (4.6%) and sepsis (2.3%) appeared more frequently in the AMS group.

### Recorded reasons for admission among patients with altered mental status, by cancer stage

**Fig. 3:**
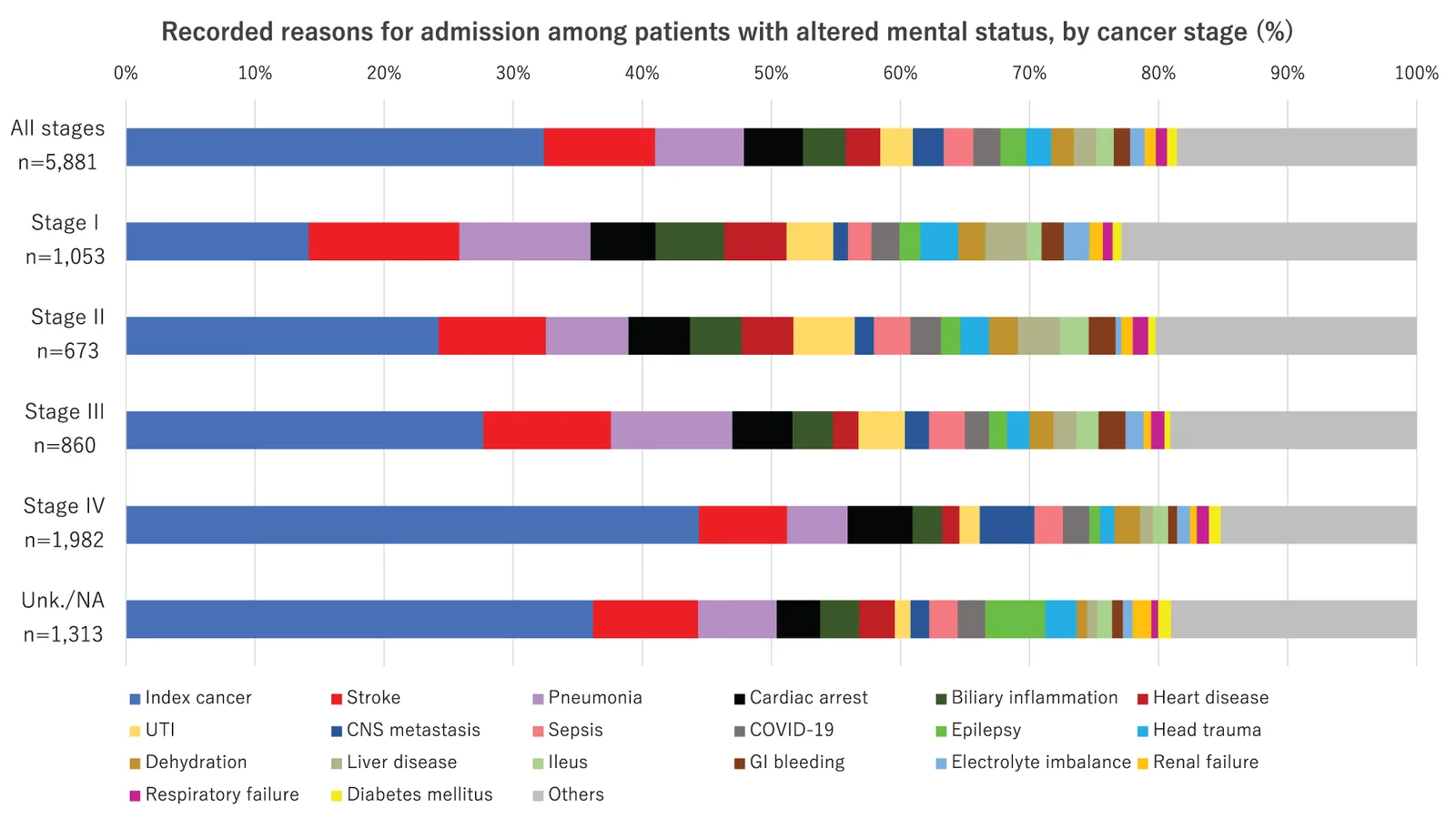
Recorded reasons for admission among patients with altered mental status, by cancer stage. CNS, central nervous system; COVID-19, coronavirus disease 2019; GI, gastrointestinal; NA, not applicable; Unk, unknown; UTI, urinary tract infection

Figure 3 illustrates the recorded reasons for admission among patients with altered mental status according to stage at cancer diagnosis. Index cancer remained the most frequent recorded reason across all stages, and its proportion increased gradually with cancer stage: 14.2% in Stage I, 23.4% in Stage II, 28.9% in Stage III, and 44.4% in Stage IV. Stroke, pneumonia, and biliary inflammation were consistently present across all stages, with similar prevalence between early and advanced stages. Sepsis and CNS metastasis were more frequent in advanced-stage cancer.

### Recorded reasons for admission among patients with altered mental status, by cancer type

**Fig. 4:**
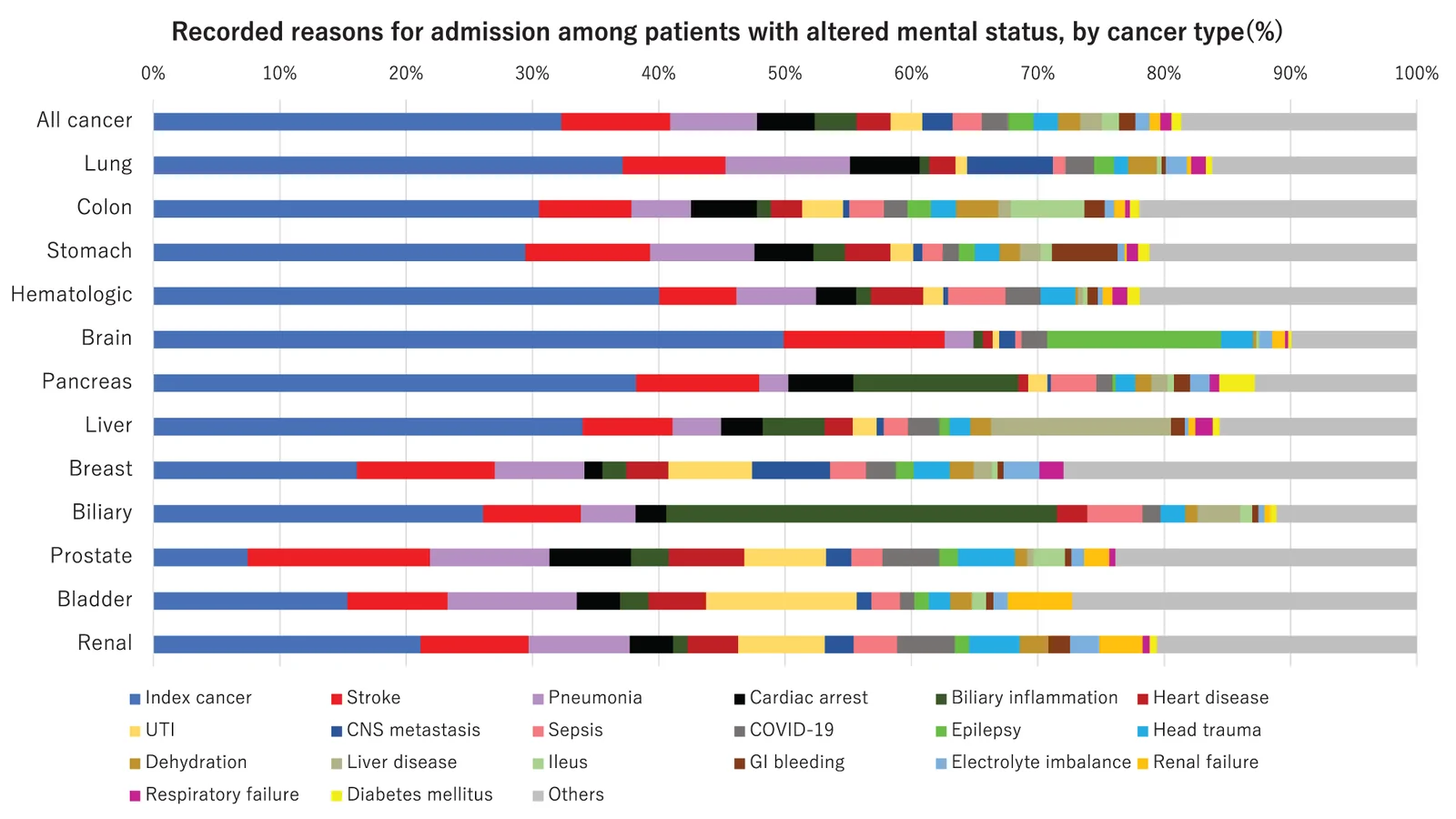
Recorded reasons for admission among patients with altered mental status, by cancer type. CNS, central nervous system; COVID-19, coronavirus disease 2019; GI, gastrointestinal; UTI, urinary tract infection

To describe the distribution of recorded reasons for admission in different cancer types, we selected the 12 most frequent cancer types in our dataset. The number of cases for each cancer type is detailed in Supplementary Table 3. For each cancer type, we identified the top seven most frequent recorded reasons for admission, ensuring that each listed diagnosis included at least five cases, as shown in Table 2. Figure 4 offers an overview of these findings across all 12 cancer types. Across the 12 major cancer types, the distribution of recorded reasons differed among malignancies. Certain reasons for admission were more frequent in specific cancers, including CNS metastasis in lung and breast cancers, pneumonia in lung cancer, sepsis in hematologic, pancreatic, and biliary cancers, urinary tract infection in prostate and bladder cancers, and hyperglycemia or diabetes mellitus in pancreatic cancer, among others.

**Table 2:** Percentages of the most frequent reasons for admissions among patients with altered mental status, by cancer type

Lung		Colon		Stomach		Hematologic	
n = 1,195		n = 806		n = 557		n = 507	
Index cancer	37.2	Index cancer	30.5	Index cancer	29.4	Index cancer	40
Pneumonia	9.9	Stroke	7.3	Stroke	9.9	Pneumonia	6.3
Stroke	8.1	Ileus	5.8	Pneumonia	8.3	Stroke	6.1
CNS metastasis	6.8	Cardiac arrest	5.2	GI bleeding	5.2	Sepsis	4.5
Cardiac arrest	5.5	Pneumonia	4.7	Cardiac arrest	4.7	Heart disease	4.1
Dehydration	2.3	Dehydration	3.4	Heart disease	3.6	Cardiac arrest	3.2
COVID-19	2.3	GI perforation	3.4	BI	2.5	Head trauma	2.8
**Brain**		**Pancreas**		**Liver**		**Breast**	
**n = 393**		**n = 390**		**n = 365**		**n = 211**	
Index cancer	49.9	Index cancer	38.2	Index cancer	34.0	Index cancer	16.1
Epilepsy	13.7	BI	13.1	Liver disease*	14.3	Stroke	10.9
Stroke	12.7	Stroke	9.7	Stroke	7.1	Pneumonia	7.1
Head trauma	2.5	Cardiac arrest	5.1	BI	4.9	UTI	6.6
Pneumonia	2.3	Sepsis	3.6	Pneumonia	3.8	CNS metastasis	6.2
COVID-19	2.0	DM	2.8	Cardiac arrest	3.3	Heart disease	3.3
Hydrocephalus	1.5	Pneumonia	2.3	COVID-19	2.5	EI	2.8
**Biliary**		**Prostate**		**Bladder**		**Renal**	
**n = 207**		**n = 201**		**n = 176**		**n = 175**	
BI	30.9	Stroke	14.4	Index cancer	15.3	Index cancer	21.1
Index cancer	26.1	Pneumonia	9.5	UTI	11.9	Stroke	8.6
Stroke	7.7	Index cancer	7.5	Pneumonia	10.2	Pneumonia	8.0
Pneumonia	4.4	UTI	6.5	Stroke	8.0	UTI	6.9
Sepsis	4.4	Cardiac arrest	6.5	Renal failure	5.1	COVID-19	4.6
Liver disease*	3.4	Heart disease	6.0	Heart disease	4.6	Head trauma	4.0
Heart disease	2.4	Head trauma	4.5	Cardiac arrest	3.4	Heart disease	4.0

*Liver disease includes hepatitis and liver failure

BI, biliary inflammation; CNS, central nervous system; COVID-19, coronavirus disease 2019; DM, diabetes mellitus; EI, electrolyte imbalance; GI, gastrointestinal; UTI, urinary tract infection

## Discussion

### Prevalence and clinical relevance of altered mental status in cancer-related emergency hospitalizations

Altered mental status was recorded in 10.5% of emergency hospitalizations after cancer diagnosis, indicating that it is a common clinical feature among cancer patients requiring emergency hospitalization. Previous studies in general emergency populations, although not directly comparable with the present study, provide useful context: altered mental status has been reported in approximately 0.9% to 5% of general ED presentations [5–9] and in 6.99% of emergency medical services (EMS)-transported patients in a Japanese population-based study using JCS criteria [22]. In the absence of directly comparable multicenter or population-based studies describing reasons for cancer-related emergency admissions involving altered mental status, our findings provide descriptive information on this clinically relevant presentation among cancer patients requiring emergency hospitalization.

### Comparison of the AMS group with the overall AEH cohort

Index cancer was the most frequent reason for admission in both the overall emergency hospitalization cohort and the AMS group. This may be because the “index cancer” category captures a broad range of admissions in which the patient’s registered primary cancer was recorded as the reason for admission, including cancer-related symptoms, functional decline, or direct complications such as poor oral intake, pain, bleeding, obstruction, respiratory compromise, or general deterioration. However, because the DPC data do not provide detailed clinical adjudication, this category may also include cases in which a more specific immediate reason for admission was not identified during emergency evaluation. Therefore, the high frequency of index cancer should be interpreted as reflecting a broad admission category rather than a single specific clinical condition.

Beyond this shared pattern, the AMS group differed from the overall emergency hospitalization cohort in several respects. Neurological and infectious diagnoses were more frequent among patients with altered mental status, including stroke (8.6%), pneumonia (6.9%), sepsis (2.3%), CNS metastasis (2.4%), and epilepsy (2.0%). Patients aged ≥ 80 years accounted for a larger proportion of the AMS group than of the AEH group. Although this observation does not determine whether age contributed to altered mental status, it aligns with prior evidence showing that older cancer patients are more likely to require emergency care and have poorer outcomes [23–25].

### Recorded reasons for admission by cancer stage

Our findings demonstrate a shift in the distribution of recorded reasons for admission depending on cancer stage at the time of diagnosis. Across all stages of cancer, stroke constituted the second most frequent reason for emergency hospitalization, after index cancer. In early-stage cancer (Stage I-II), metabolic and infectious diagnoses such as biliary inflammation and pneumonia were more prevalent, whereas in advanced-stage cancer (Stage III-IV), index cancer and CNS metastasis accounted for larger proportions of emergency admissions. These patterns are compatible with the clinical expectation that cancer progression and metastatic complications are more frequently encountered in later-stage disease. Sepsis was more frequently recorded in Stages II-IV than in Stage I. This pattern is clinically plausible, as patients with more advanced cancer may be more vulnerable to systemic infection because of disease-related factors and treatment-related immunosuppression [26, 27]. However, treatment details and immune status were not assessed in the present study.

### Recorded reasons for admission by cancer type

The distribution of recorded reasons for admission differed by cancer type, and several patterns were clinically plausible in light of known complications of specific malignancies.

Among neurological diagnoses, epilepsy and hydrocephalus were frequently recorded in patients with brain tumors, while CNS metastasis was more frequently recorded in lung and breast cancers compared to other malignancies. This pattern is consistent with previous studies showing that lung and breast cancers are common primary malignancies associated with brain metastases [28–31]. Stroke accounted for 6–14% of admission diagnoses across various cancer types, with relatively high percentages observed in brain tumor, prostate cancer, and breast cancer.

Among infectious diagnoses, pneumonia was frequently recorded in lung cancer, but also remained a common reason for admission across most cancer types. Biliary inflammation was common in pancreatic, biliary, and liver cancers, while UTIs were frequent in prostate, renal, and bladder cancers. Sepsis was relatively frequent in hematologic, pancreatic, and biliary cancers. These patterns are consistent with the anatomical and clinical characteristics of these malignancies.

Several other admission diagnoses also appeared to cluster by cancer type. Hyperglycemia/diabetes mellitus was frequently recorded in pancreatic cancer, ileus in colon cancer, and gastrointestinal bleeding in stomach cancer; these patterns are clinically recognizable and are supported by previous literature on cancer-type-specific complications [32–34].

These patterns suggest that the admission diagnoses observed among patients with altered mental status are not uniform across cancer types, but instead broadly align with known complication profiles of the underlying malignancies. Taken together, these descriptive findings may inform clinical awareness of cancer-specific patterns among patients presenting with altered mental status in emergency settings.

### Limitations

Despite the strengths of using a large multicenter cancer registry, our study has some limitations. First, the analysis relied on administrative data, including the DPC “reason for admission” field. Therefore, the coded reason for admission cannot be assumed to represent the confirmed etiology of altered mental status in each patient, and differences by cancer stage or cancer type should not be interpreted as causal relationships. For example, patients recorded as being admitted for index cancer, pneumonia, stroke, or other diagnoses may have had altered mental status through multiple overlapping mechanisms.

Second, altered mental status was defined as JCS ≥ 1. Although JCS is widely used in Japan and routinely recorded in the DPC system, this threshold captures a broad clinical spectrum, ranging from mild confusion or disorientation to severe impairment of consciousness. Disorientation in patients with dementia may also have led to inclusion in the AMS group, resulting in a broader definition than that used in some previous studies. Furthermore, because the DPC system records JCS but not GCS, direct comparison with international studies that predominantly use GCS may be limited.

Third, this study focused on emergency hospitalizations and did not account for patients who were evaluated in the ED and discharged home. Future studies including non-hospitalized ED patients could provide a more comprehensive picture of altered mental status in cancer-related emergency care.

## Conclusion

This multicenter registry-based study showed that altered mental status was recorded in 10.5% of first emergency hospitalizations after cancer diagnosis. Among patients with altered mental status, the recorded reasons for admission varied according to cancer stage and cancer type, with index cancer, stroke, pneumonia, CNS metastasis, sepsis, and other neurological or infectious diagnoses frequently observed. These descriptive findings may inform clinical awareness of cancer-specific patterns among patients presenting with altered mental status in emergency settings. Future research is needed to confirm these findings and to explore approaches to improve outcomes for cancer patients presenting with altered mental status.

## Electronic supplementary material

Below is the link to the electronic supplementary material.

Supplementary Material 1

## Data Availability

The data supporting this study’s findings can be obtained from the corresponding author upon reasonable request.

## Abbreviations

AEH: All Emergency Hospitalizations
AMS: Altered Mental Status
CanReCO: Cancer Registry-based Study on Cancer Care in Osaka
CNS: Central Nervous System
COVID-19: Coronavirus Disease 2019
DPC: Diagnosis Procedure Combination
ED: Emergency Department
GI: Gastrointestinal
GCS: Glasgow Coma Scale
ICD: International Classification of Diseases
JCS: Japan Coma Scale
UTI: Urinary Tract Infection
