# Supplementary material for: Altered mental status in cancer-related emergency hospitalizations: prevalence and recorded reasons for admission
Source: BMC Emerg Med. 2026 Jul 6;26:263. doi: 10.1186/s12873-026-01667-9 (PMC13647859; doi:10.1186/s12873-026-01667-9)
Supplement: Supplementary file 1 [file 12873_2026_1667_MOESM1_ESM.docx]

**Supplement Table 1. The assignment of codes for cancer types**

| **Cancer type** | **ICD-O-3^a^** | **ICD-O-3^a^ morphology** |
| --- | --- | --- |
| Oral | C00-C14 |  |
| Esophagus | C15 |  |
| Stomach | C16 |  |
| Colon | C18-C20 |  |
| Liver | C22 |  |
| Biliary | C23, C24 |  |
| Pancreas | C25 |  |
| Larynx | C32 |  |
| Lung | C33, C34 |  |
| Bone | C40, C41, C47, C49 |  |
| Skin | C43, C44 |  |
| Breast | C50 |  |
| Uterus | C53-C55 |  |
| Ovary | C56 |  |
| Prostate | C61 |  |
| Bladder | C67 |  |
| Renal | C64-C66, C68 |  |
| Brain | C700, C71, C751-753 |  |
| Thyroid | C73 |  |
| Hematologic tumors |  | 959-976, 980-998 |
| Other malignant neoplasms | Others |  |

^a^ International Classification of Diseases for Oncology, Third edition

**Supplement Table 2. The assignment of codes for reasons for admission**

| **Reason for admission** | **ICD-10^a^** |
| --- | --- |
| Agranulocytosis | D70 |
| Alzheimer's disease | G30 |
| Aortic dissection or aneurysm | I71 |
| Biliary inflammation | K80, K81, K83 |
| Cardiac arrest | I46 |
| Chronic obstructive pulmonary disease | J41-J43 |
| CNS metastasis | C793 |
| COVID-19 | U07 |
| Dehydration | E86 |
| Diabetes mellitus | E10-E14 |
| Electrolyte imbalance | E87 |
| Epilepsy | G40, G41 |
| Externally caused injuries | V01-X59, Y10-Y34 |
| Gastrointestinal bleeding | K92 |
| Gastrointestinal perforation | K63 |
| Head trauma | S06 |
| Heart disease | I00-I09, I11, I13, I20-52 |
| Ileus | K56 |
| Interstitial pneumonia | J84 |
| Liver disease | K70-K76 |
| Peritonitis | K65 |
| Pneumonia | J12-J18, J69 |
| Postoperative complications | T81 |
| Renal failure | N17-N19 |
| Senility | R54 |
| Sepsis | A40, A41 |
| Stroke | I60-I69 |
| Suicide | X60-X84, Y87 |
| UTI | N10, N11, N39 |
| Other diseases | A00-U99, not in the above |

^a^ International Classification of Diseases, Tenth revision

CNS, central nervous system; COVID-19, Coronavirus Disease 2019; UTI, urinary tract infection

**Supplement Table 3. Number of emergency hospitalizations with altered mental status by cancer type**

| **Cancer type** | **n** |
| --- | --- |
| Lung | 1195 |
| Colon | 806 |
| Stomach | 557 |
| Hematologic | 507 |
| Brain | 393 |
| Pancreas | 390 |
| Liver | 365 |
| Breast | 211 |
| Others | 208 |
| Biliary | 207 |
| Prostate | 201 |
| Bladder | 176 |
| Renal | 175 |
| Esophagus | 136 |
| Oral | 104 |
| Skin | 80 |
| Uterus | 74 |
| Ovary | 39 |
| Larynx | 25 |
| Thyroid | 17 |
| Bone | 15 |
